# Herpes Zoster Infection in an Immunocompromised Patient: A Case Report and Review of Corticosteroid’s Role

**DOI:** 10.7759/cureus.20908

**Published:** 2022-01-03

**Authors:** Rui Seixas, Filipe Dias, Armindo Ribeiro, Sofia Sobral, Henrique Rita

**Affiliations:** 1 Internal Medicine, Unidade Local de Saúde do Litoral Alentejano, Santiago do Cacém, PRT; 2 Internal Medicine, Hospital Do Litoral Alentejano, Santiago do Cacém, PRT

**Keywords:** immunosuppression, viral infection, corticosteroid treatment, immunocompromised patient, herpes zoster virus

## Abstract

Herpes zoster infection is more frequent and severe in the immunocompromised population. Its incidence is significantly higher in this population when compared to immunocompetent individuals. The authors present a case of an 88-year-old man with a history of arterial hypertension and myelodysplastic syndrome. The patient was evaluated in the emergency department (ED) for edema of the frontal facial region with left periorbital involvement and multiple purulent vesicles. He was diagnosed with viral infection by herpes zoster and was prescribed valaciclovir and deflazacort. He returned two days later due to an increase in the lesions affecting the left hemi-cranium, with decreased visual acuity and painless purulent drainage in the ipsilateral eye. The ophthalmological evaluation revealed exuberant edema with associated chemosis and involvement of the cornea of ​​the affected eye. He was hospitalized and began antiviral therapy with intravenous acyclovir and chloramphenicol with topical ganciclovir. There was a progressive resolution of the skin lesions but no reversal of the loss of vision in the affected eye. Herpes zoster infection is more frequent and severe in the immunocompromised population. The clinical presentation is often similar to that of the immunocompetent population; however, it can have exuberant manifestations. The authors emphasize the need for close clinical monitoring of the immunocompromised patient with herpes zoster infection and review the role of corticosteroids when treating this particular population.

## Introduction

Herpes zoster is caused by the varicella-zoster virus (VZV), which after initial infection, remains latent in posterior root ganglia of the human spinal cord and can be reactivated in the presence of multiple factors, such as advanced age, physical trauma, psychological stress, cancer, radiotherapy, or states of immunosuppression [1]. Reactivation of the infection typically manifests as a painful rash accompanied by blisters involving a specific dermatome. Patients infected with herpes zoster usually report a history of childhood chickenpox, neuropathic pain along a dermatome, prodrome, fever, headache, and itching or burning sensation in the affected area [2]. Herpes zoster infection is more frequent and severe in the immunocompromised population. Its incidence is significantly higher in this population when compared to immunocompetent individuals over the age of 60 years [3]. The clinical presentation is often similar; however, it can have exuberant manifestations [4]. The main objective in the management of these patients is to reduce the incidence of cutaneous and visceral involvement, associated with a high risk of mortality [5]. Its treatment consists of an antiviral regimen, which depends on the extent of the infection and the presence or absence of an immunosuppression state [6].

## Case presentation

**Figure 1:**
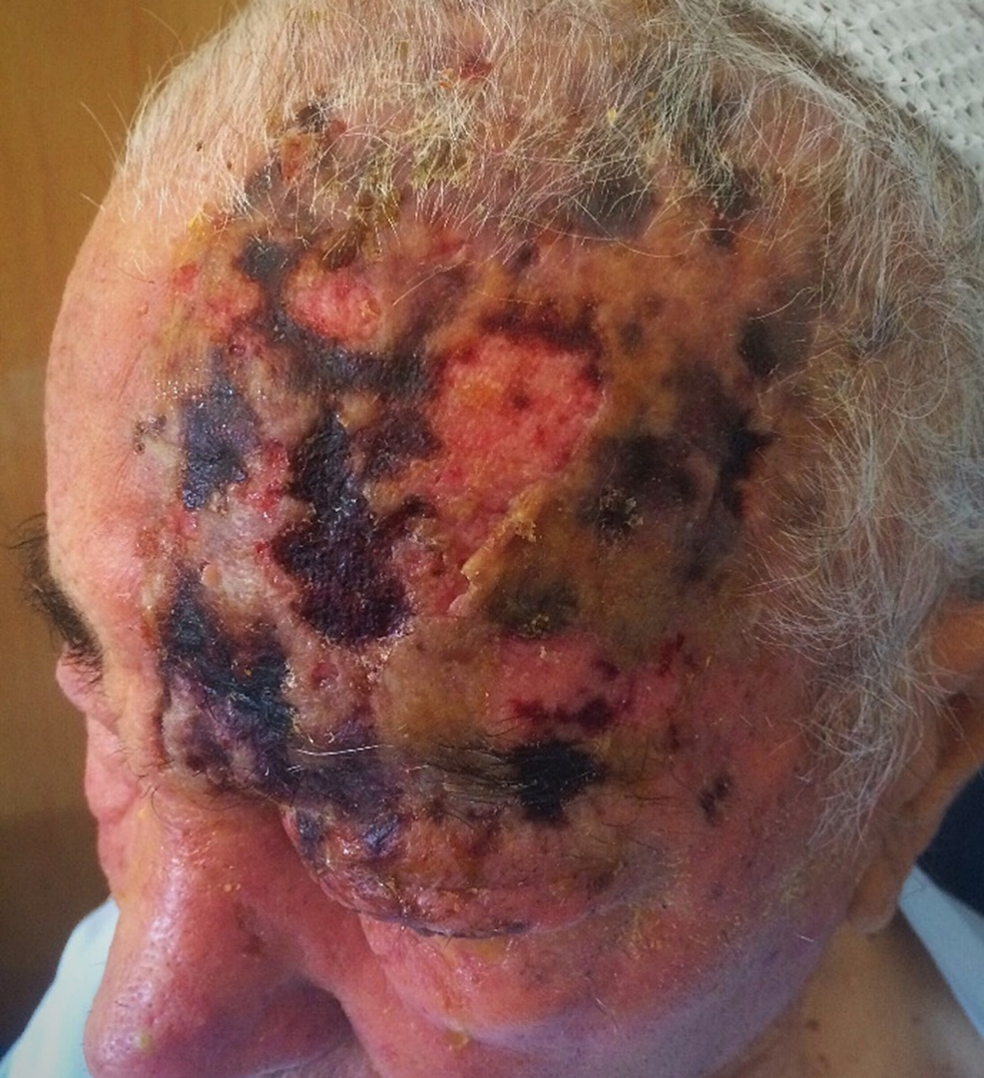
Infection by herpes zoster with the frontal facial region and left periorbital involvement (before treatment).

The authors present a case of an 88-year-old man with a history of arterial hypertension and newly diagnosed myelodysplastic syndrome. The patient was evaluated in the emergency department (ED) for edema of the frontal facial region with left periorbital involvement and multiple purulent vesicles that had started one week before his presentation. He was diagnosed with a viral infection by herpes zoster and was discharged with a prescription of valaciclovir and deflazacort. He came back two days later due to worsening of the lesions affecting the left hemi-cranium, new-onset decreased visual acuity, and painless purulent drainage in the ipsilateral eye (Figure 1).

**Figure 2:**
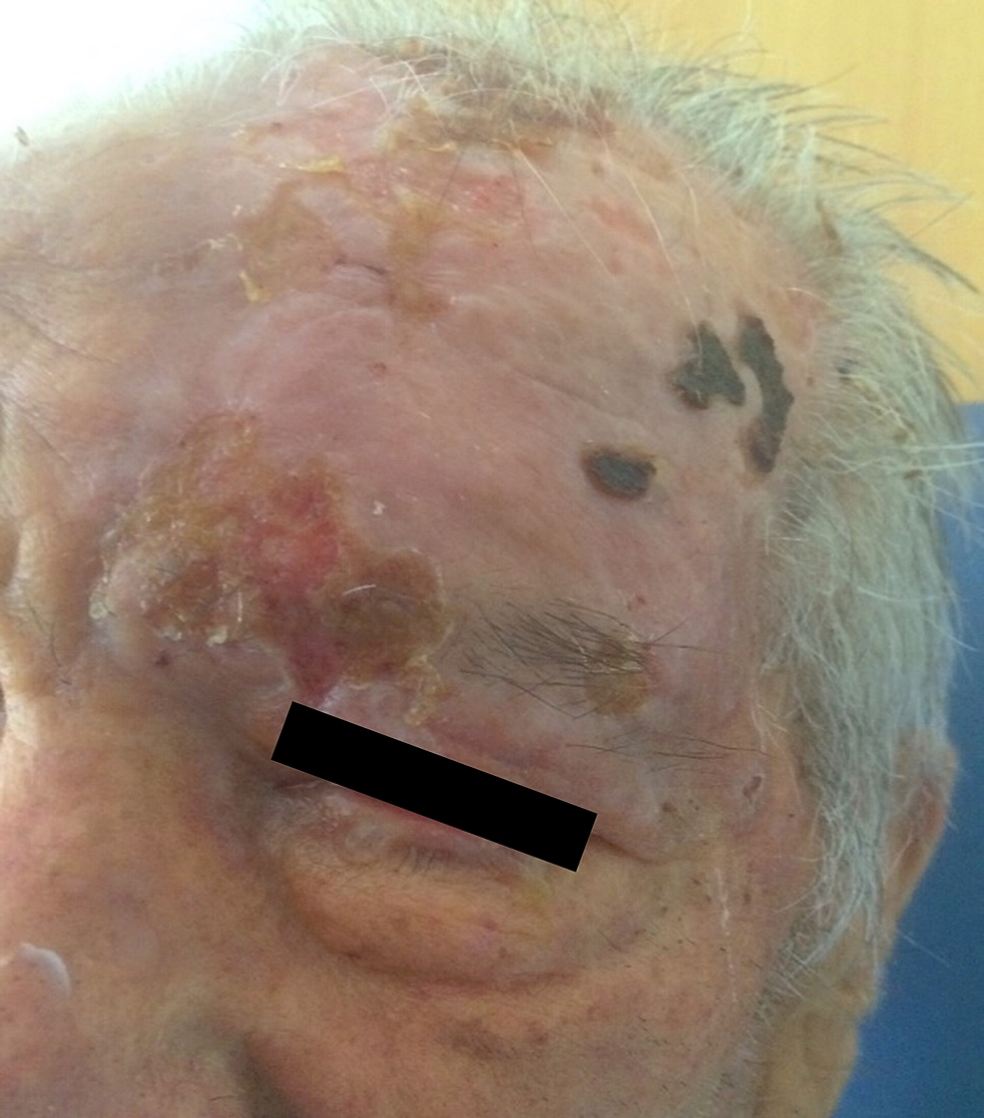
Infection by herpes zoster post-treatment.

The ophthalmologic evaluation revealed exuberant edema with associated chemosis and involvement of the cornea of ​​the affected eye. The remaining clinical evaluation was unremarkable and no neurological symptoms were identified. His bloodwork revealed pancytopenia (hemoglobin: 7.3 g/dL, platelets: 79 x 109/L, and white blood cells: 2.9 x 109/L) and slight elevation of C-reactive protein (4.2 mg/L; normal range < 3.0 mg/L). Chest X-ray showed no alterations. The patient was hospitalized with antiviral therapy with intravenous acyclovir and chloramphenicol as well as topical ganciclovir. There was a progressive resolution of the skin lesions; however, no reversal of the loss of vision in the left eye was noted, which evolved to complete blindness (Figure 2).

The remaining period of hospitalization elapsed with no complications. Three weeks later, the patient was discharged with a referral to ophthalmology and internal medicine consultation.

## Discussion

Although most cases of herpes zoster infection are self-limited, early symptomatic and antiviral therapy is associated with a significant reduction in morbidity as well as time to resolution [7]. After resolution of the herpes zoster Infection, many patients report a chronic pain state, designated as postherpetic neuralgia, most common after the involvement of the trigeminal region and in patients over the age of 55 and characterized by pain in the affected region with a typical duration of 1 to 3 months, but which may in some cases take years to resolve. [8]. Other complications include encephalitis, bacterial superinfections, skin lesions, retinal necrosis causing blindness, and cerebral ataxia, among others [9]. Zoster ophthalmicus also carries an increased risk of transient ischemic attack and stroke. Even though in the case described by the authors, there was an identified cause for immunosuppression (an underlying myelodysplastic syndrome), it is important to bear in mind that in patients without an identified cause for immunosuppression, VZV reactivation can be the first manifestation of an undiagnosed neoplasm, infection by HIV, or other immunosuppressive states, which is why etiological study must always be performed [10]. The use of corticosteroids in immunosuppressed patients is dubious, with the literature showing either conflicting results or includes recommendations based on clinical experience and not clinical trials [11]. It has been recommended as adjuvant therapy in accelerating the resolution of acute neuritis [12] but no role was identified in the prevention and control of postherpetic neuralgia [13]. However, recent studies have shown that the prolonged use of corticosteroids in the geriatric population can increase the risk of developing herpes zoster due to its immunosuppressing effects [14]. The authors question the use of corticosteroids in this previous immunocompromised patient as a possible trigger for a rapid progression and severe presentation of the infection. Official recommendations for the management of the herpes zoster infection state that the use of corticosteroids in the immunocompetent population, debatable as it may be, has shown several benefits in reducing acute pain and improving the quality of life, and it does not increase the risk of dissemination [15]. However, when dealing with a fragile population such as geriatric patients and even more so in those who are immunocompromised, the boundary between benefits and complications becomes more blurry with no recommendation stated in the guidelines for the management of this disease [16,17]. Thus, larger randomized studies in these populations are needed.

## Conclusions

Herpes zoster infection can appear in a weakened immune system and can be associated with several complications, which is why patients should be studied and followed up to manage any sequelae of the infection and detect any immunocompromising underlying conditions. Treatment with corticosteroids is still debatable, but its use in the immunocompromised patient, if done, should be monitored with extreme care due to the risk of a further worsening of the viral infection with systemic dissemination.
